# [Retracted] Isoflurane reduces pain and inhibits apoptosis of myocardial cells through the phosphoinositide 3-kinase/protein kinase B signaling pathway in mice during cardiac surgery

**DOI:** 10.3892/mmr.2025.13579

**Published:** 2025-05-27

**Authors:** Zhibing Pi, Hai Lin, Jianping Yang

Mol Med Rep 17: 6497–6505, 2018; DOI: 10.3892/mmr.2018.8642

Following the publication of the above paper, it was drawn to the Editor's attention by a concerned reader that the flow cytometric assay data shown in Fig. 16 on p. 6053 had already appeared in an article written by different authors at a different research institute that was published previously in the journal *Biomed Research International*.

Owing to the fact that the contentious data in the above article had already been published prior to its submission to *Molecular Medicine Reports*, the Editor has decided that this paper should be retracted from the Journal. The authors were asked for an explanation to account for these concerns, but the Editorial Office did not receive a reply. The Editor apologizes to the readership for any inconvenience caused.

